# Case Report: Coexistence of a giant borderline phyllodes tumor of the breast and a contralateral fibroadenoma: a challenging case with clinical manifestations and PET-CT imaging mimicking advanced cancer

**DOI:** 10.3389/fonc.2026.1793170

**Published:** 2026-03-17

**Authors:** Xin-Ru Wang, Qiang Zhang, Xing-Guo Cui

**Affiliations:** 1 Department of Breast and Thyroid Surgery, the Affiliated Hospital of Yanbian University (Yanbian Hospital), Yanji, Jilin, China; 2 Department of Medical Imaging and Nuclear Medicine, the Affiliated Hospital of Yanbian University (Yanbian Hospital), Yanji, Jilin, China

**Keywords:** borderline phyllodes tumor, breast tumor, fibroadenoma, multidisciplinary diagnosis and treatment, PET-CT

## Abstract

**Background:**

Phyllodes Tumor (PT) is a rare fibroepithelial tumor, which is classified into three categories: benign, borderline, and malignant. The three types show a gradually increasing trend in terms of local recurrence and distant metastasis. Giant PT usually refers to a mass with a diameter larger than 10 cm, accounting for approximately 20% of PT cases. Borderline PT (BPT) has overlapping clinical manifestations with both benign fibroadenomas and malignant breast tumors. Especially when it presents with rapid growth and ulceration, it is easily misdiagnosed as advanced breast cancer.

**Case presentation:**

This paper reports a case of a 44-year-old female patient who was admitted to the hospital with the chief complaint of “discovering a painless mass in the left breast several months ago and presenting due to rapid enlargement accompanied by skin ulceration in the past month”. Physical examination revealed a huge mass (approximately 24×17×15 cm) in the left breast, with diffuse skin edema, redness, increased skin temperature, and multiple skin ulcers.A small nodule can be palpated in the right breast. Preoperative auxiliary examinations: Breast ultrasound indicated a nodule in the right breast (BI-RADS category 4a) and disordered glandular structure in the left breast (BI-RADS category 0) accompanied by axillary lymph node enlargement; PET-CT showed significantly increased metabolism in the left breast mass (SUVmax 11.5) and ipsilateral axillary lymph nodes (SUVmax 9.0), highly suggestive of malignancy with axillary lymph node metastasis. Core needle biopsy (CNB) of the left breast mass suggested a fibroepithelial tumor, favoring PT, but the grade could not be determined. After discussion by the multidisciplinary team (MDT), the patient underwent total mastectomy of the left breast + axillary lymph node dissection(ALND) of the left side + resection of the right breast mass. Postoperative paraffin pathology and extensive immunohistochemistry confirmed BPT of the left breast (mitotic figures approximately 7/10 HPF, negative surgical margins), and all 21 dissected lymph nodes on the ipsilateral side showed reactive hyperplasia; the nodule in the right breast was a FA.

**Conclusion:**

This case highlights the diagnostic dilemma of giant BPT, which clinically and radiologically mimics locally advanced or metastatic breast cancer. Preoperative CNB has limitations in PT grading, and the final diagnosis depends on histopathological examination of completely resected specimens. For tumors with necrosis, ulceration, and infection, the high metabolic findings on PET-CT should be interpreted with caution, as they may be caused by secondary inflammatory responses rather than specifically indicating highly invasive cancer or metastasis, thus avoiding overstaging and overtreatment before surgery. The core of treatment is wide surgical resection with negative margins, and a prudent approach should be taken towards axillary management, with routine axillary dissection not recommended. The multidisciplinary collaborative model is crucial for integrating conflicting information and achieving individualized and precise decision-making. In the future, it is necessary to explore a dynamic risk assessment system integrating clinical, pathological, and molecular features to improve the individualized management level of PT.

## 1 Introduction

PT is a rare fibroepithelial tumor originating from the mesenchymal tissue in the terminal duct lobular unit of the breast, accounting for approximately 0.3% - 1% of breast tumors (1). Giant PT, typically referring to masses with a diameter exceeding 10 cm, accounts for about 20% of PTs (2). They are most commonly found in women aged 45 to 50 years and are often unilateral. Histologically, this tumor features a characteristic “phyllodes” structure, presenting as a biphasic proliferation of epithelial and stromal components (3). It has a wide spectrum of biological behaviors, which can evolve from a benign growth pattern to highly invasive sarcomatous transformation (4). Therefore, accurate pathological diagnosis and grading are crucial for clinical prognosis assessment. Currently, WHO classifies PT into three categories: benign, borderline, and malignant, based on multiple histological indicators such as stromal cell density, cellular atypia, number of mitotic figures, tumor margin status, and the presence of stromal overgrowth. These three types of tumors show significant differences in clinical prognosis.Despite the clear WHO criteria, there are significant differences between the actual pathological assessment and the clinical diagnostic results (5), partly because its biological behavior is difficult to predict, and histological classification does not always accurately reflect clinical behavior. To reduce diagnostic disparities and assist clinical decision-making, the academic community is seeking more objective histological scoring methods and auxiliary detection tools. From 2023 to 2025, our hospital performed approximately 1836 breast mass resection surgeries, among which 13 cases were pathologically diagnosed as PT.However, there was only one case of giant PT.

When a BPT presents as a large mass with rapid growth, accompanied by skin ulceration, necrosis, and secondary infection, its clinical and imaging features can be easily confused with those of advanced breast cancer.Although PET-CT can be used to evaluate the metabolic activity of tumors, the infiltration of inflammatory cells caused by internal necrosis, hemorrhage, and secondary infection in tumors can lead to a significant increase in local glucose metabolism, presenting as high ^18^F-FDG uptake on PET-CT (6). Attributing the hypermetabolic phenomenon solely to the highly invasive behavior or metastasis of malignant tumors may lead to misjudgment of tumor biological behavior and over - staging may lead to misjudgment of the tumor’s biological behavior and overstaging (7).Meanwhile, CNB has a small sampling volume and limited spatial coverage, making it difficult to comprehensively reflect the molecular and pathological characteristics of the entire tumor, resulting in limited accuracy of grading. Especially in highly heterogeneous tumors such as PT, the accuracy of preoperative pathological grading is often affected (8). Ultimately, the diagnosis highly depends on the histopathological and immunohistochemical evaluation of completely resected specimens. Therefore, Integrate multimodal information and postoperative pathological information to develop individualized treatment strategies under the collaboration of a MDT has become the key in the current diagnosis and treatment of PT.This paper reports a rare case of a giant ulcerated BPT in the left breast accompanied by a FA in the contralateral breast. Preoperative PET-CT indicated a malignant tumor with axillary lymph node metastasis, while preoperative CNB only suggested a “phyllodes tumor tendency”. Through the MDT collaboration, the patient received standardized surgical treatment. The postoperative pathology finally confirmed a left breast borderline phyllodes tumor accompanied by extensive necrosis and infection, with no axillary lymph node metastasis, and the right breast presented with a FA. This article reveals the diagnostic challenges of giant BPTs and serves as a warning against unnecessary axillary lymph node dissection.

## 2 Case presentation

### 2.1 Clinical data

A 44-year-old female patient was admitted to the hospital on May 18, 2025, due to “an asymptomatic mass in the left breast was accidentally discovered several months ago, and the mass rapidly enlarged in the past month, accompanied by tenderness and skin ulceration”. The patient had been in good health previously, and there was no family history of chronic diseases or malignancies. Her menstrual cycle was regular. Her marriage and child-bearing history: She was married and had one daughter.

### 2.2 Physical examination

**Figure 1:**
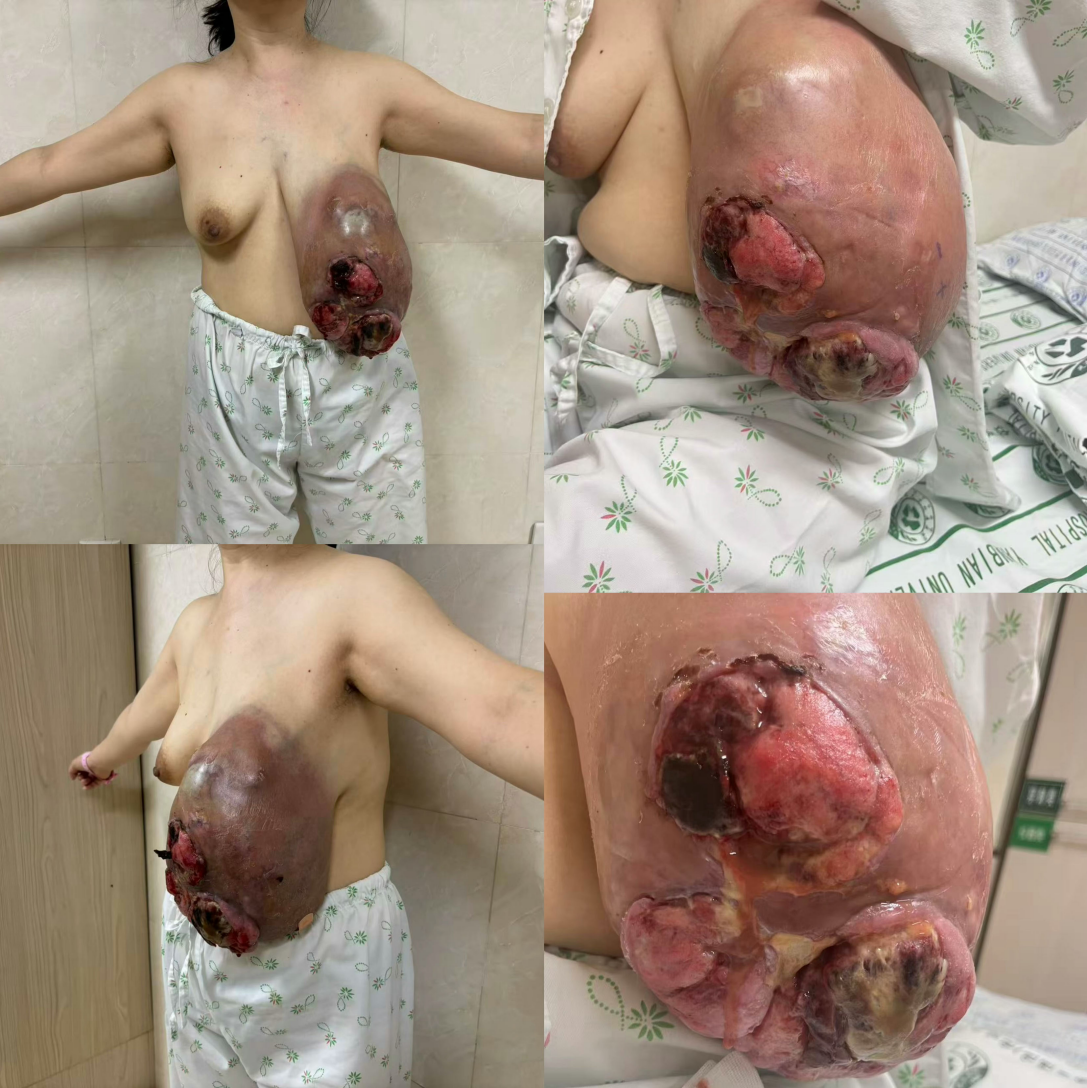
Preoperative clinical photograph.

There was obvious asymmetry between the bilateral breasts. The left breast was significantly enlarged, showing diffuse edema, red and swollen skin, and increased skin temperature. Three skin ulcers were visible on the surface, with sizes of 10x7 cm, 10x7 cm, and 5x5 cm respectively. A huge mass of approximately 24x17x15 cm could be palpated in the left breast, which was hard in texture, with unclear boundaries, poor mobility, tenderness, and was fixed to the skin but not adhered to the underlying pectoralis major muscle. The structure of the nipple and areola of the left breast was difficult to identify. The right breast appeared normal, and a mass of approximately 2.0×1.5 cm was palpated beside the nipple at the 12 o’clock position, which was tough in texture, with clear boundaries and good mobility. No obvious enlarged lymph nodes were palpable in the bilateral axillae and supraclavicular regions (**Figure 1**).

### 2.3 Auxiliary examinations

**Figure 2:**
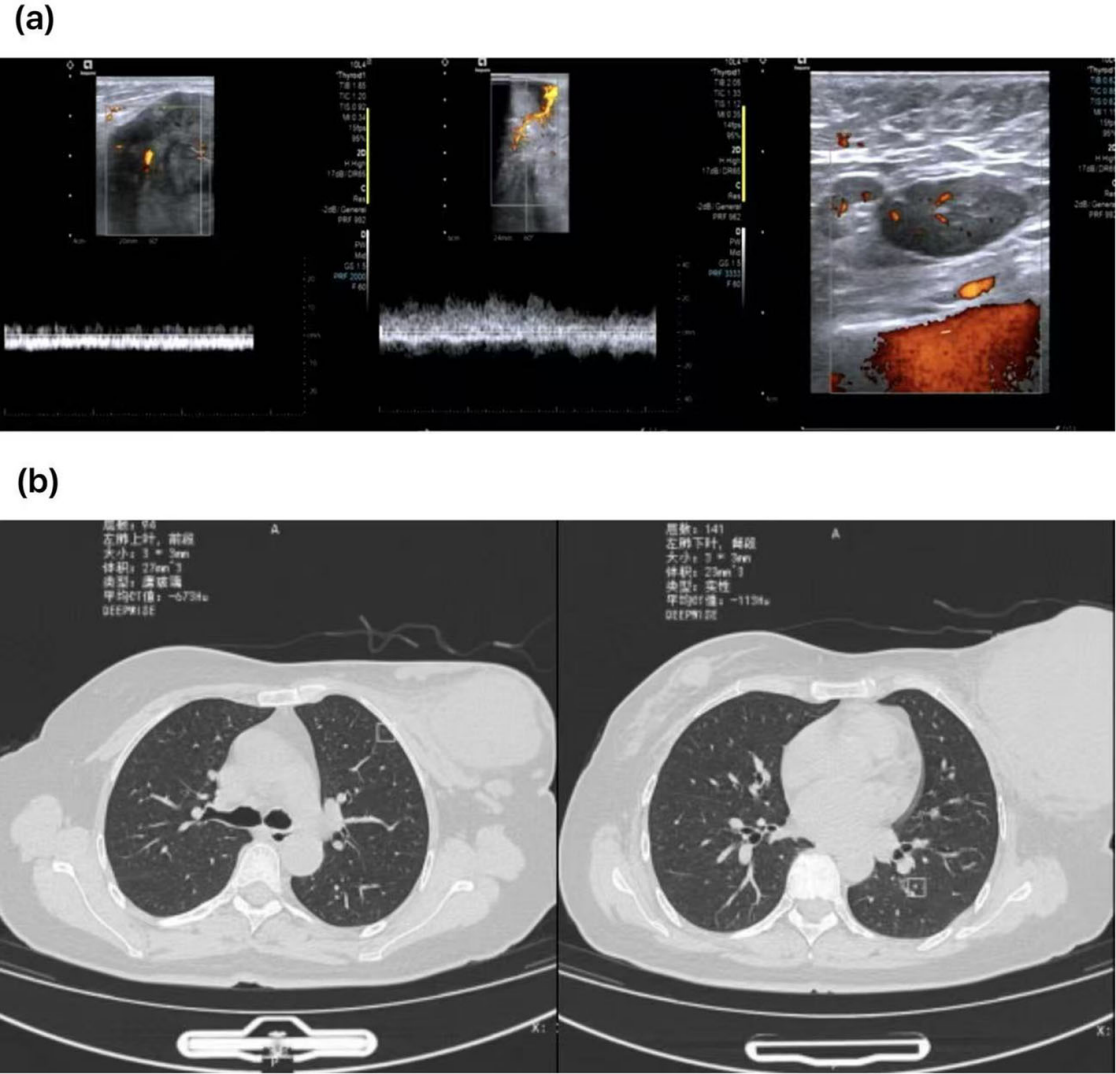
**(A)** Preoperative ultrasound revealed unclear architecture and abundant blood flow in the left breast.Enlarged left axillary lymph node. **(B)** A giant soft tissue mass was identified in the left breast, with a small nodule in the right breast. Multiple scattered nodules were noted in both lungs; a low-density shadow was present in the left inner lobe of the liver; and splenomegaly was observed.

Ultrasonography: Nodule in the right breast (BI-RADS category 4a). The glandular structure of the left breast is unclear, showing mixed echoes, and CDFI indicates rich blood flow (BI-RADS category 0); The demarcation between the cortex and medulla of the left axillary lymph nodes is unclear (**Figure 2A**).

Chest CT: A huge soft tissue mass in the left breast and a nodule in the right breast. Scattered small nodules in both lungs; A low-density shadow in the left medial lobe of the liver; Splenomegaly (**Figure 2B**).

**Figure 3:**
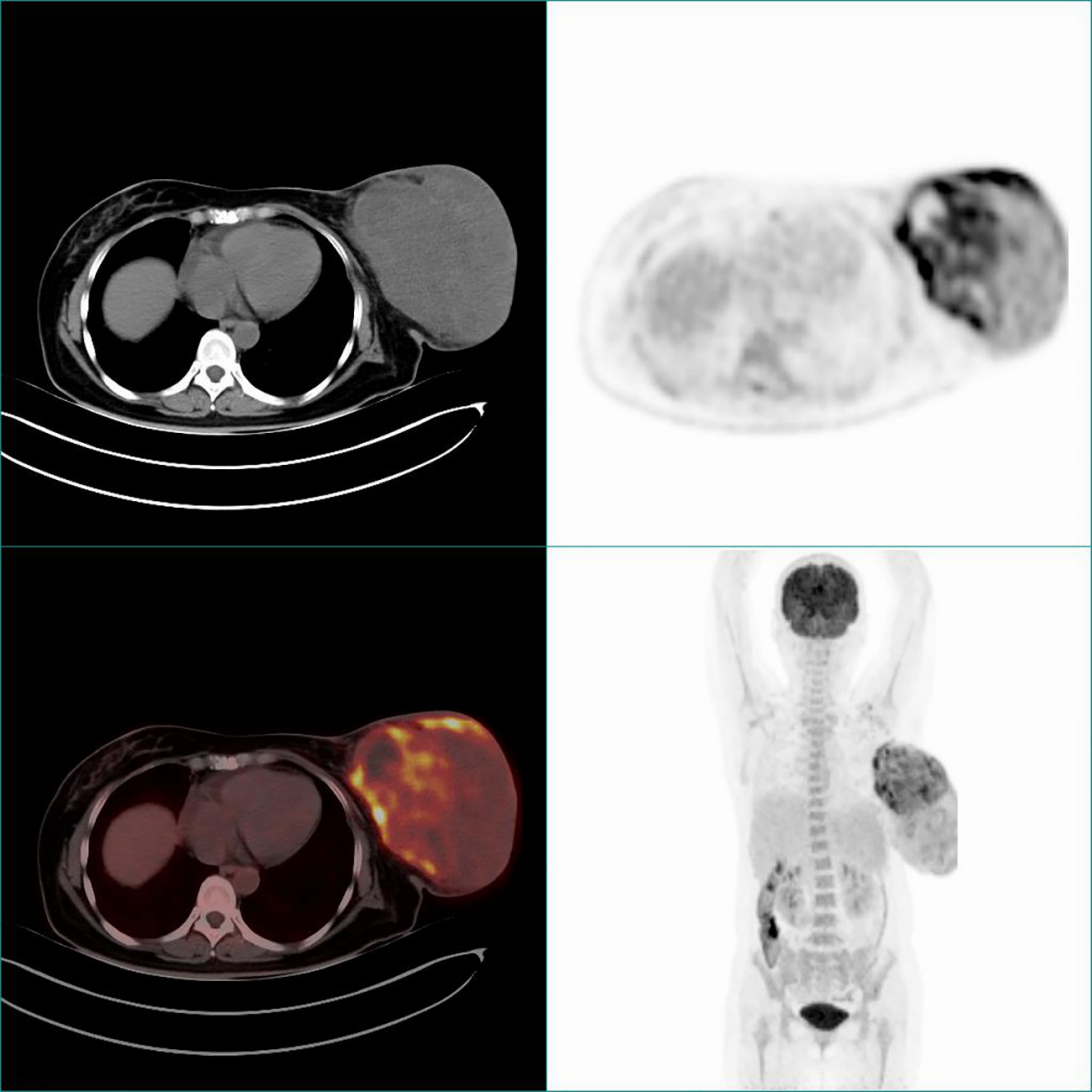
Preoperative PET-CT images.

PET-CT: The metabolism of the left breast mass (SUVmax 11.5) and the ipsilateral axillary lymph nodes (SUVmax 9.0) was significantly increased. It suggests the possibility of malignant tumor with lymph node metastasis; the metabolism of the right breast nodule was mildly increased (SUVmax 1.7). The metabolism of the small nodules in both lungs and the lesion in the left medial lobe of the liver was not high (**Figure 3**).

The following abnormal results were observed in the blood test: the tumor markers CA15 - 3, CA19 - 9, and CA - 125 were all mildly elevated, and the inflammatory indicators such as C - reactive protein and serum amyloid A were significantly elevated. A hemoglobin level of 93 g/L indicated mild anemia in the patient.

Preoperative pathology (CNB of the left breast): Microscopically, a fibroepithelial tumor was observed, favoring PT, accompanied by papillary hyperplasia of ductal epithelium. Immunohistochemistry (IHC) showed CK5/6(+), P63(+), and a low stromal Ki-67 index (approximately 2%). Due to the limited specimen, the benign or malignant grading could not be determined.

### 2.4 MDT discussion and surgical treatment/diagnosis and treatment decision-making

An MDT discussion was organized on May 21, 2025. The core opinions are summarized as follows:

Department of Pathology: The preoperative puncture specimens are limited, and the stromal atypia of the tumor is not obvious, making it difficult to grade the tumor before surgery. The final diagnosis depends on completely resected specimens.

Department of Nuclear Medicine: High metabolism on PET-CT requires differentiation between tumor activity and inflammatory response. High metabolism of axillary lymph nodes is considered metastatic.However, the high metabolic consequences caused by inflammation need to be vigilant.

Department of Radiology: The micronodules in both lungs show no obvious increase in metabolism, and the possibility of metastasis is low.

Department of Medical Oncology/Radiation Oncology: It is recommended to perform surgery first, and then determine the adjuvant treatment strategy based on the final pathology.

MDT consensus: The patient has a huge tumor in the left breast, which grows rapidly and invades the skin, with clear surgical indications. The nature of the nodule in the right breast needs to be confirmed by intraoperative frozen section. The treatment goal is to completely resect the left breast tumor and ensure negative margins. Given that the preoperative PET-CT shows increased metabolism of the ipsilateral axillary lymph nodes, in order to clarify the staging, ALND is planned to be performed simultaneously.

### 2.5 Surgery and pathology

On May 22, 2025, the patient underwent right breast mass resection, left mastectomy, and left ALND. The patient underwent surgical treatment under general anesthesia. The patient first underwent resection of the right breast mass. The pathological examination indicated a fibroepithelial tumor, with a tendency towards FA. Subsequently, left mastectomy and left ALND (Levels I and II) were carried out. The surgical design for the left breast was a radical incision including the skin ulceration area and the nipple. The margin of the incision was approximately 3 cm from the tumor edge. The long thoracic nerve and thoracodorsal nerve were preserved during the operation, and no invasion of the pectoralis major muscle was found. The intraoperative blood loss was approximately 20 ml, and the procedure was smooth.

**Figure 4:**
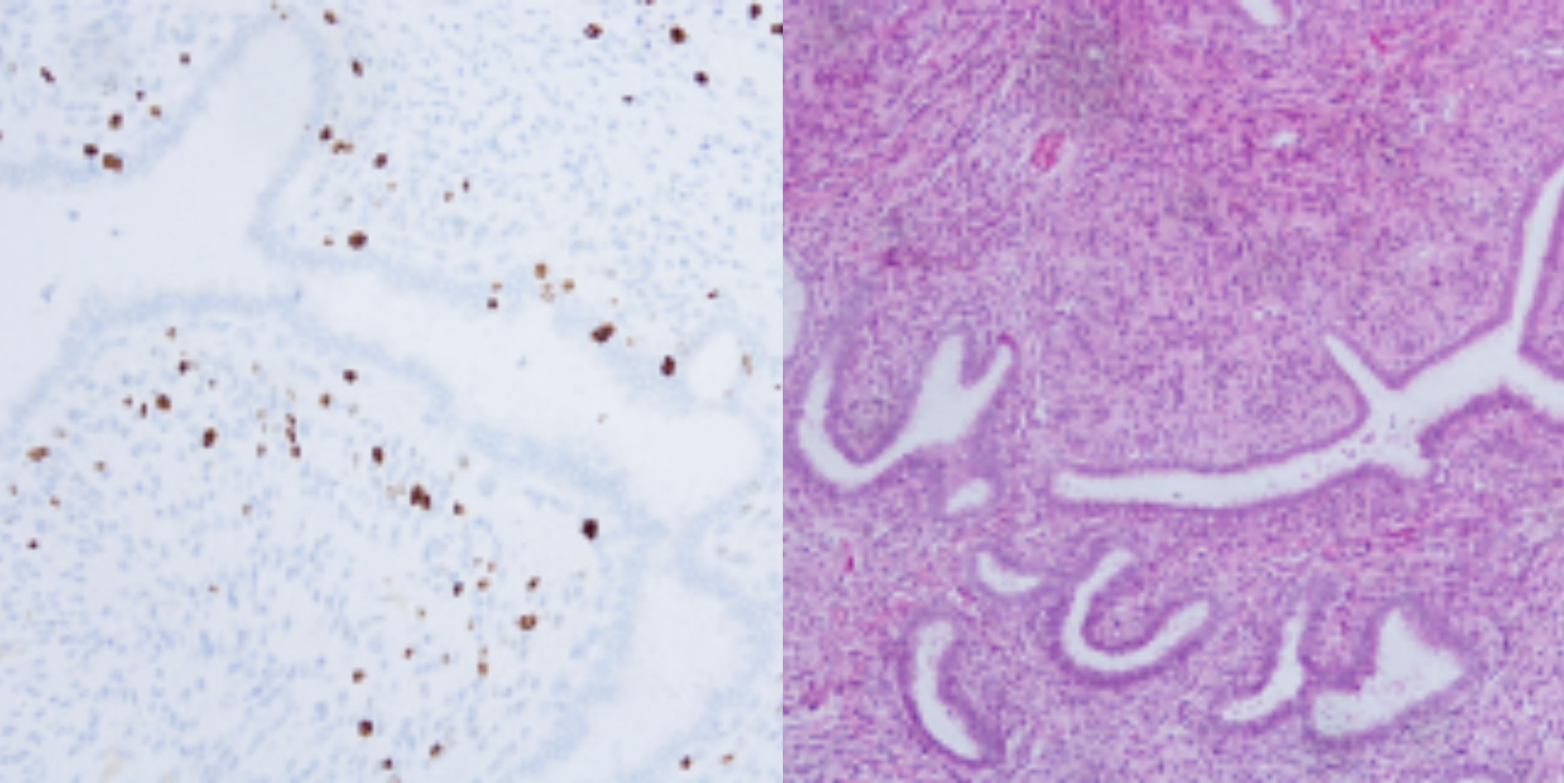
Pathological images (H&E staining).

Postoperative pathology further confirmed the diagnosis. The paraffin section of the left breast was consistent with a BPT: Microscopically, it showed a characteristic phyllodes structure, with approximately 7 mitotic figures per 10 high-power fields (HPF). The tumor had extensive necrosis and secondary infection, involving the dermis but not the nipple. All surgical margins were negative (>1 cm). Immunohistochemistry revealed CK (glandular +), CK5/6 (focal +), P63 (focal +), Ki-67 (approximately 5% + in the hotspots) and p53 (focal weak +) (**Figure 4**). All 21 lymph nodes removed from the left axillary dissection showed reactive hyperplasia, and no tumor metastasis was observed. The mass in the right breast is a FA, and the surgical margins are clear.

### 2.6 Postoperative course/recovery and follow-up

The patient recovered well after the operation. The drainage tubes were gradually removed, and the patient was discharged on the 12th day after the operation. Due to the borderline histology, negative surgical margins, and no lymph node metastasis, adjuvant radiotherapy was postponed after discussion by the MDT.

**Figure 5:**
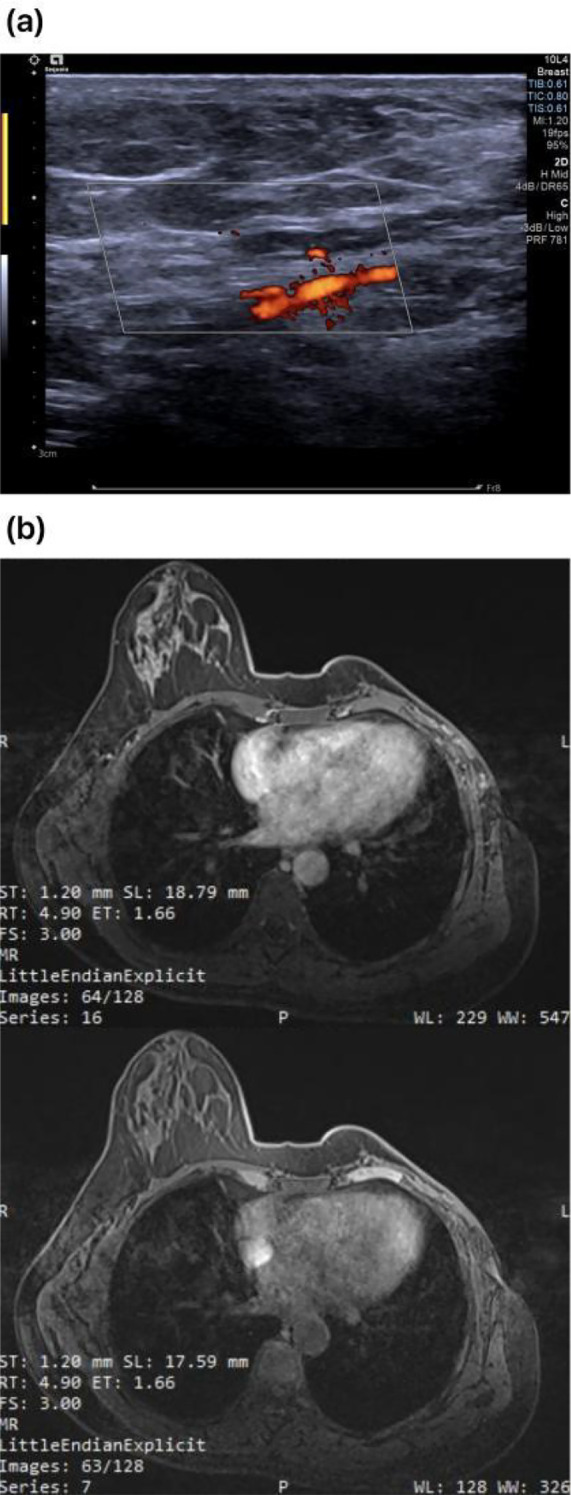
Follow-up images of breast color Doppler ultrasound **(A)** and breast MRI **(B)**.

The patient is undergoing a follow - up consultation. A follow - up plan is formulated. Within 2 years after the operation, clinical physical examinations and imaging examinations (breast/chest wall ultrasound, chest CT) are to be conducted every 6 months, aiming to detect local recurrence or distant metastasis at an early stage. PTs have a relatively high tendency for local recurrence. The patient’s condition was stable at the time of discharge.Six months after discharge, the patient underwent a follow - up breast color Doppler ultrasound and magnetic resonance imaging at our hospital (**Figure 5**).

## 3 Discussion

This case comprehensively demonstrates the profound diagnostic and therapeutic challenges faced by BPT in clinical practice. Particularly when it presents with rapid growth, a large size, and skin ulceration, it can be easily confused with locally advanced breast cancer.

### 3.1 Prognostic features of BPTs: between benign and malignant

PT of the breast is closely related to histological grade. First, regarding the differences in 5 - year survival rates among benign, borderline, and malignant PTs, multiple studies have provided specific data support. Patients with benign PTs have an excellent prognosis, while the 5 - year survival rate of patients with malignant PTs is significantly reduced (9). Second, the data on local recurrence rates have also been verified by multiple studies. The status of surgical margins is a key factor affecting local recurrence, and the histological grade is positively correlated with the recurrence risk (10). The local recurrence rate of malignant PTs is significantly higher than that of benign PTs (11). The incidence of distant metastasis in malignant PTs ranges from 9% to 27% (12). The distant metastasis rate of BPTs is relatively low, but it can still occur. It is particularly alarming that local recurrence is often accompanied by an upgrade in the biological behavior of the tumor, with the risk of transformation to a higher - grade malignancy (13). Although this case is a BPT, it presents with invasive clinical features such as a large size, skin ulceration, and extensive necrosis. Its biological behavior has exceeded the typical borderline category, suggesting the limitations of the traditional grading system.

### 3.2 Pitfalls of PET-CT in necrotizing infected PTs: inflammation-induced high FDG uptake

In this case, the pre - operative PET - CT showed remarkable FDG hypermetabolism in both the left breast mass and axillary lymph nodes, strongly suggesting breast cancer with lymph node metastasis. Post - operative pathology revealed that this hypermetabolic phenomenon was caused by the inflammatory response triggered by extensive internal tumor necrosis and infection, rather than the biological activity of the malignant tumor itself; the ipsilateral axillary lymph nodes also showed high metabolic signs due to inflammatory cell infiltration (14). Studies have shown that activated inflammatory cells in the tumor microenvironment have high glycolytic activity, which can lead to significant high FDG uptake (15). In addition, large tumors are often accompanied by the “mass effect” and secondary inflammation, which further exacerbates their high-metabolism pattern on PET-CT, similar to that of highly invasive malignant tumors (16). This case suggests that in the evaluation of such complex lesions, high metabolism in regional lymph nodes does not necessarily indicate tumor metastasis. Currently, imaging strategies for distinguishing inflammatory and neoplastic FDG uptake are still in the exploratory stage. Dual-time-point imaging, SUV pattern analysis, and correlation studies with serum inflammatory markers have all shown certain potential, but there is a lack of standardized protocols. Novel tracers such as fibroblast activation protein inhibitors (FAPI) are expected to reduce the interference of inflammatory background and improve the accuracy of tumor detection due to their high expression in tumor-associated fibroblasts and low uptake in inflammatory cells (17). In the future, prospective studies are needed to evaluate the diagnostic efficacy of FAPI in fibroepithelial lesions and to explore multimodal integration models of PET radiomics with ultrasound and pathological features.

### 3.3 Limitations of CNB in the grading of PTs and the importance of surgical margins

Due to the high intratumoral heterogeneity of PT itself, the limited puncture tissue may miss the lesion area with the most active mitotic figures or the most atypical features (18). Currently, the academic community generally believes that the core value of CNB lies in clarifying the fibroepithelial nature of the lesion and initially excluding breast cancer. Nevertheless, it has significant limitations in the accurate grading of PT, mainly because the small sampling volume and insufficient spatial coverage of CNB make it difficult to fully represent the pathological and molecular characteristics of the tumor (19). Therefore, the final diagnosis depends on a systematic pathological evaluation of the completely resected specimen.Surgical resection is the core of BPT treatment. The status of surgical margins has become one of the key factors affecting prognosis (20). To reduce the risk of recurrence, it is currently widely believed that a negative surgical margin of ≥1 cm should be achieved (21). In this case, the tumor is extremely large and has invaded the skin. Therefore, total mastectomy is a reasonable choice (22).

### 3.4 Warnings for axillary treatment: follow guidelines to avoid unnecessary ALND

This case is a warning case resulting from the interpretation of PET-CT results, highlighting the fundamental difference between PT and breast cancer in the principle of lymph node management.As a tumor originating from mesenchymal tissues, PT mainly metastasizes through the bloodstream, and lymph node metastasis is relatively rare, accounting for only 1.1% to 3.8% (23). Based on this pathophysiological characteristic, mainstream international guidelines do not recommend routine ALND or sentinel lymph node biopsy (SLNB) as a standard component of BPT treatment (24). Excessive axillary surgery not only fails to improve the survival prognosis but also significantly increases the risk of postoperative complications, such as lymphedema, sensory and motor dysfunction of the upper limb, and limited shoulder joint mobility, which seriously affect the patient’s quality of life. In this case, ALND was performed based on the “positive” lymph nodes indicated by PET - CT. Postoperative pathology confirmed that all 21 lymph nodes showed reactive hyperplasia without evidence of metastasis.This cautionary experience strongly validates the rationality of the current guideline recommendations: For clinically or radiologically suspicious lymph nodes, priority should be given to obtaining pathological confirmation through puncture biopsy or SLNB (25), rather than directly performing radical lymph node dissection.This strategy of “diagnosis first, then treatment” can not only ensure oncology safety but also maximize the protection of the patient’s function and quality of life. We present this case as a teaching example with the aim of reminding our colleagues to avoid making the same mistakes.

### 3.5 The spectrum relationship between FA and PT: molecular evidence and clinical implications

Both PT and FA of the breast belong to fibroepithelial lesions.The clinical coexistence phenomenon suggests that the two might be in different evolutionary stages of the same disease spectrum.Molecular pathological studies provide strong support for this. FA and benign or BPT share key early genetic alterations, especially somatic mutations in the MED12 gene, which occur in up to 60%–80% (26) of FAs and are also frequently detected in PTs (27), indicating that the mutation may be the common genetic basis for this type of fibroepithelial lesion. Research has confirmed that there is a clear trajectory of cumulative genetic mutations from FA to benign, borderline, and then to malignant PT (28). The simultaneous occurrence of FA and PT in both breasts may reflect the patient’s overall breast tissue susceptibility to specific molecular abnormalities or the differentiation of a common precursor clone into different phenotypes under different microenvironments (29). In this case, the simultaneous occurrence of BPT and FA, although uncommon, is not accidental. This phenomenon alerts the clinic that when FA is found on one side, the entire breast should be systematically evaluated to rule out potential PT lesions.

### 3.6 Moving towards risk stratification models: beyond traditional morphological classification

The traditional PT classification system that solely relies on histological morphology has obvious limitations, especially in its insufficient ability to predict the recurrence risk of patients in the intermediate-risk group. Some PTs histologically defined as “benign” may still experience local recurrence (30), and the clinical outcomes of “borderline” cases are highly heterogeneous, with their biological behavior presenting a continuous spectrum (31). Simply relying on histological morphology for static tumor classification has obvious limitations. In this case, the patient’s invasive clinical course, such as a large tumor size and skin ulceration, did not fully match the “borderline” histological diagnosis, further highlighting the inadequacy of the current grading system. This phenomenon is highly similar to the development of diagnosis and treatment of gastrointestinal stromal tumors (GIST). In the early stage, GIST was also classified simply as benign or malignant, but now it has fully shifted to a risk stratification model integrating multiple parameters (32) such as tumor size, mitotic count, location, and gene mutation status (33). These models divide patients into very low-, low-, intermediate-, and high-risk groups, significantly improving the accuracy of prognosis prediction and the pertinence of treatment decisions (34). Therefore, for PT, a type of tumors with heterogeneous clinical behaviors, the successful experience of GIST should be learned in the future to construct a multi-dimensional information risk assessment system integrating clinical, pathological, molecular, and imaging data. The variables recommended for inclusion are as follows: 1. Clinical parameters: maximum tumor diameter, growth rate, and skin invasion; 2. Pathological parameters: margin status (35), mitotic count, excessive stromal growth, necrosis extent, and cellular atypia; 3. Molecular markers: MED12 mutation status, TERT promoter mutation, and chromosomal copy number variation; 4. Imaging features: PET-CT metabolic parameters (SUVmax, MTV, TLG), dynamic contrast-enhanced MRI features, and ultrasound radiomics features. This model can be prospectively validated through multicenter studies, standardized data collection, and external validation. It is expected to break through the bottleneck of traditional morphological classification and achieve more precise individualized management.

### 3.7 Limitations of this case

As a single-case report, this study has the following limitations: 1. The imaging-pathology correlation requires validation with more cases; 2. Management decisions are significantly influenced by the MDT judgments of this center and may not be applicable to all clinical settings; 3. The follow-up time is relatively short; 4. Molecular testing was not conducted to verify the spectrum relationship between FA and PT. However, this case provides an important warning for understanding the imaging pitfalls of BPT and the principles of axillary management.

## 4 Conclusion

This case highlights the diagnostic challenges posed by giant BPT mimicking advanced breast cancer. Postoperative confirmation revealed that the high metabolism on PET - CT and lymph node signs of this case were due to the inflammatory response caused by tumor necrosis and infection, which warns clinicians to interpret the imaging results of such masses with caution.The diagnosis and treatment experience further clarifies the limitations of CNB in preoperative grading of PTs and emphasizes that pathological evaluation after complete surgical resection is the recognized standard for the diagnosis and grading of PT. The core objective of surgical treatment should be to obtain a sufficient negative surgical margin. In this case, axillary lymph node dissection was performed based on positive PET-CT results, and postoperative examination confirmed that all 21 lymph nodes showed reactive hyperplasia. This experience emphasizes the need to strictly follow the guideline recommendations that ALND is not routinely performed for PT.For suspicious lymph nodes, puncture biopsy or SLNB should be prioritized over direct lymph node dissection.At a deeper level, the coexistence of FA and PT in this case suggests a potential association between the two within the spectrum of fibroepithelial lesions, and shared genetic bases such as MED12 mutations provide molecular evidence for this. In the future, shifting the management strategy from traditional morphological classification to a dynamic risk stratification model integrating clinical, pathological, and molecular features is the key direction for achieving precise and individualized treatment.

## Data Availability

The raw data supporting the conclusions of this article will be made available by the authors, without undue reservation.
